# The relevance of prelamin A and RAD51 as molecular biomarkers in cervical cancer

**DOI:** 10.18632/oncotarget.21686

**Published:** 2017-10-09

**Authors:** Simona Leonardi, Marianna Buttarelli, Ilaria De Stefano, Gabriella Ferrandina, Marco Petrillo, Gabriele Babini, Giovanni Scambia, Carmela Marino, Mariateresa Mancuso, Daniela Gallo

**Affiliations:** ^1^ Department of Sustainability, Agenzia Nazionale per le Nuove Tecnologie, l’Energia e lo Sviluppo Economico Sostenibile (ENEA), Rome, Italy; ^2^ Department of Obstetrics and Gynecology, Catholic University of the Sacred Heart, Rome, Italy; ^3^ Department of Radiation Physics, Guglielmo Marconi University, Rome, Italy; ^4^ Department of Physics, University of Pavia, Pavia, Italy

**Keywords:** cervix, LACC, chemoradiotherapy, DNA repair, lamin A/C

## Abstract

Along with their role in the maintenance of nuclear architecture, nuclear lamins also control genomic stability, DNA damage repair, transcription, cell proliferation, differentiation and senescence. Recent reports reveal that prelamin A–processing defects play a role in cancer development by impacting on transcription of key players in the maintenance of the genome stability, including RAD51. Here, we performed a ‘proof of concept’ study evaluating the role of prelamin A and RAD51 expression in clinical outcome of cervical cancer patients. We analyzed biomarker expression by immunohistochemistry in tumor material from locally advanced cervical cancer (LACC) patients (n=66) and correlated data with clinicopathological parameters and with response to neoadjuvant chemoradiation (CT/RT). In LACC patients who underwent neoadjuvant CT/RT the percentage of cases showing high prelamin A levels was greater in patients who completely responded to treatment (25 of 40, 62.5%) than in patients with macroscopic residual tumor (6 of 26, 23.1%, p=0.0024). Conversely, patients showing high RAD51 expression were less likely to respond to treatment (14 of 26, 53.8%) than were those with low protein levels (12 of 40, 30%, p=0.072). Only prelamin A retained an independent role in predicting response to treatment (p=0.003), while RAD51 approached statistical significance (p=0.07). Notably, high RAD51 expression highly significantly predicted poor outcome, emerging as an independent prognostic factor for disease free survival (p=0.038), while approaching statistical significance for overall survival (p=0.09). Our findings provide a framework for future prospective studies investigating molecular predictors of response to neoadjuvant chemoradiotherapy in LACC patients.

## INTRODUCTION

Cervical cancer is the fourth most common cancer in women and the seventh overall, with an estimated 528,000 new cases and 266,000 deaths in 2012, thus accounting for 7.5% of all cancer deaths in females [1]. Nearly all cervical cancers are caused by *human papilloma virus* (HPV) and just two HPV types, 16 and 18, are responsible for about 70% of all cases [2]. While early lesions are cured with surgery or radiation (RT) alone, the standard treatment for LACC patients (FIGO stage IB2 through stage IVA) is concurrent chemoradiotherapy (CT/RT) [3, 4], being cisplatin the most commonly used drug, either alone or in combination with other agents (e.g. 5-fluorouracil [5-FU] or hydroxyurea). Although concurrent chemoradiation has significantly improved disease control and survival, patients in advanced stage of cervical cancer are at greater risk of recurrence and account for the majority of deaths, with a 5-year overall survival of about 70% [5–7]. In this context, investigational approaches using either neoadjuvant chemotherapy (NACT) or CT/RT followed by radical surgery (RS) have reported encouraging results in terms of clinical outcome, with acceptable toxicity [8–13]. Moreover, two randomized studies aimed at assessing the efficacy of completion surgery after CT/RT versus CT/RT only showed that CT/RT followed by RS is not superior to exclusive CT/RT in spite of different toxicity profile [14, 15]. Notably, as imaging techniques have been shown not to be able to accurately detect residual tumor after neoadjuvant approaches [16], completion surgery after chemoradiation actually represents the only method to reliably obtain the most important prognostic information, that is, pathological response to treatment [8–10]. Pathological assessment of response to treatment might indeed have clinically relevant implications for definition of risk and pattern of recurrence, individualized patient counselling, and choice to administer adjuvant treatment. Clinicopathologic factors, including stage and tumor histology, as well as sophisticated imaging techniques like MRI and PET-CT scan may serve as markers for responsiveness to radiotherapy, but they are not likely to fully account for the observed variability. For this reason, various microarray studies have been performed in advanced-stage cervical cancer patients to identify biological markers predictive of response to radiotherapy, but no definitive results have been reached yet [17, 18]. According to recently published data, EGFR signaling, C-erbB-2, and COX-2 could represent interesting indicators of poor response to chemoradiation [7], but unfortunately, to date, no one single biomarker has achieved combined sensitivity and specificity values across the breadth of clinical presentations.

The A-type lamins, lamin A/C belongs to type V intermediate filaments and together with B-type lamins, form the nuclear lamina in most somatic mammalian cells. At the cellular level, both classes of proteins have been ascribed structural roles in the nucleus as well as a range of other activities, including coordination of transcription and replication [19]. Prelamin A, the normal translation product of *LMNA* mRNA, is post-translationally processed into lamin A by two transfer reactions and two proteolytic cleavages [20]. Prelamin A-processing defects cause a series of human diseases, collectively called laminopathies, the most severe of which are Hutchinson Gilford progeria syndrome (HGPS) and restrictive dermopathy (RD), caused by prelamin A, or by truncated forms of prelamin A accumulation, respectively [21]. HGPS and RD cells accumulate, with passage in culture, endogenous DNA damage, in particular DSBs (double-strand breaks), indicating that DNA repair activity is impaired in these cells [20]. Given the functional relevance of the DNA repair system on carcinogenesis, several recent reports have revealed that loss of lamin A/C expression represents one of the molecular alterations that contribute to the acquisition of hallmark traits of cancer, and indeed lamin A/C deficiency has been linked to the development of different tumors [22]. Relevant to our interest, an extensive study was performed on the expression of lamins in normal cervical epithelium and in premalignant lesions (CIN, cervical intra-epithelia neoplasia), results showing a prominent lamin A staining of the entire epithelium, with a general decrease in high grade CIN lesions [23]. Moreover, prelamin A has been identified as a member of the “central core of cervical cancer” [24]. Notably, defective DNA repair arising from lamin A/C-deficiency, has been recognized as one of the factors that underlie sensitivity to DNA damaging agents, and actually, the laminopathy-based progeroid cells were found to be sensitive to various DNA-damaging agents, including DSB inducers (ionizing radiation, camptothecin, and etoposide), mitomycin C (inducing interstrand cross-links), and the alkylating agent, methyl methanesulfonate [20]. Among the protein regulated by A-type lamins, Rb family members, 53BP1, BRCA1 and RAD51 are known to play key roles in DNA DSBs repair by NHEJ (Non-homologous end joining) and HR (homologous recombination); consequently, in the context of defective maturation of prelamin A, loss of these proteins critically contributes to increased sensitivity to radiation or chemotherapy [25]. On the other hand, previous studies have demonstrated that HPV E7 increases RAD51 (and BRCA1) levels in a transcriptional and potentially posttranscriptional manner [26], with a downregulation in cervical tumour samples following chemoradiotherapy [27].

Taking into account all these findings, we performed a ‘proof of concept’ study testing the impact of defective DNA repair arising from defective maturation of prelamin A, with reduction of RAD51, on treatment response and disease outcome in patients with LACC.

## RESULTS

A total of 66 LACC patients, 5 histopathologically normal cervical samples, 5 low-grade and 5 high-grade squamous intraepithelial lesions (low-SIL and high-SIL, respectively) were evaluated, by immunohistochemical analysis, for prelamin A and RAD51 expression. In normal cervical tissues and in low-SILs results showed high prelamin A (mean score ± SEM 3.8 ± 0.2 for both) and low RAD51 (mean score 1.2 ± 0.2 and 1.2 ± 0.4, respectively) expression levels; on the other hand, high-SIL exhibited much lower prelamin A expression (mean score 1.8 ± 0.2), without a significant change in RAD51 levels (mean score 1.3 ± 0.1) (Supplementary Figure 1). In the entire cancer series, prelamin A and RAD51 levels were 2.2 ± 0.14 and 2.4 ± 0.12 (mean ± SEM). For prelamin A, samples with high expression levels mostly showed strong staining at the nuclear rim of tumor cells, while low-expression cases typically showed a punctate cytoplasmic staining. RAD51 exhibited instead a more diffuse staining pattern in both the cytoplasm and nucleus as expected in a pre-treatment tumor biopsy (i.e. before exposure to radiation, no-stress condition), although cases with high expression levels more often displayed RAD51 foci; this latter feature is in line with previous reports showing that when RAD51 is overexpressed, foci and higher order structures are seen even in the absence of induced DNA damage and even when cells are not in S phase [28].

### Correlation with clinicopathological parameters

**Figure 1:**
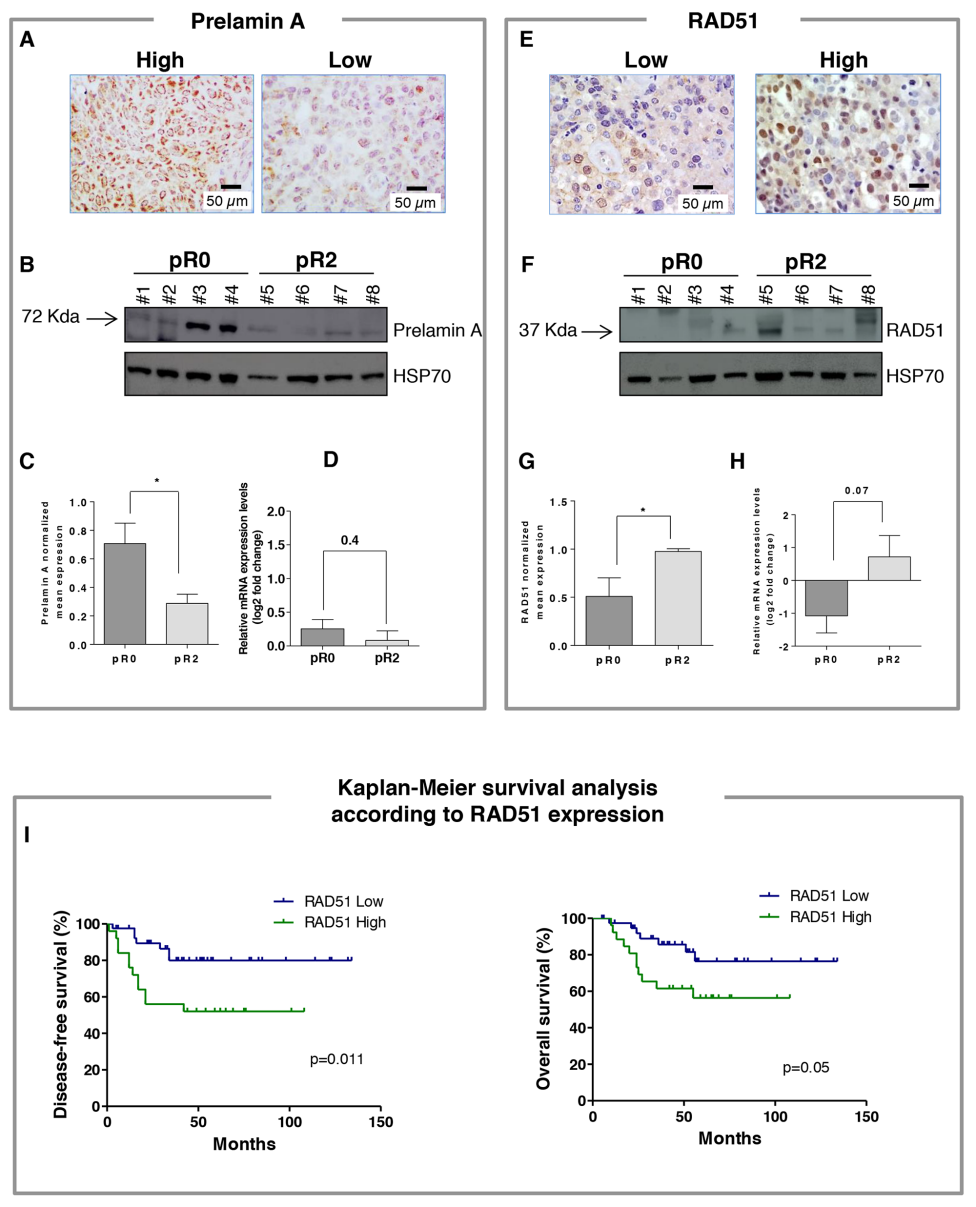
**(A)** Representative pictures for high and low prelamin A immunostaining in locally advanced cervical cancer (LACC) patients (magnification 40x). **(B)** Representative western blot analysis of prelamin A expressions from patients who completely responded to treatment (pR0) and patients with macroscopic residual tumor (pR2) after chemoradiotherapy. #No.= patient code. **(C)** Densitometric analysis of normalized prelamin A levels respect to HSP70; columns represent the mean ± SEM for each group (n=10 for both groups). Difference was statistically significant between tumors from pR0 and pR2 groups (*p* < 0.05). **(D)** The relative mRNA expression of *LMNA* was evaluated by RT-PCR, utilizing specific set of primers. All samples were normalized to the housekeeping gene, *B2M*. Results are presented as the mean ± SEM of log2 fold change representative of mRNA expression levels relative to the whole population of samples (n=10 for both groups). **(E)** Representative pictures for low and high RAD51 immunostaining in LACC patients (magnification 40x). **(F)** Representative western blot analysis of RAD51 expressions from patients who completely responded to treatment (pR0) and patients with macroscopic residual tumor (pR2) after chemoradiotherapy. #No.= patient code. **(G)** Densitometric analysis of normalized RAD51 levels respect to HSP70; columns represent the mean ± SEM for each group (n=10 for both groups). Difference was statistically significant between tumors from pR0 and pR2 groups (*p* < 0.05). **(H)** The relative mRNA expression of *RAD51* was evaluated by RT-PCR, utilizing specific set of primers. All samples were normalized to the housekeeping gene, *B2M*. Results are presented as the mean ± SEM of log2 fold change representative of mRNA expression levels relative to the whole population of samples (n=10 for both groups). **(I)** Kaplan–Meier survival curve for the probability of disease-free survival (left) and overall survival (right) according to expression of RAD51 in LACC patients. High expression of RAD51 was significantly associated with disease-free survival and overall survival disadvantage (*p* = 0.011 and *p* = 0.05, respectively).

The distribution of cases with high prelamin A and high RAD51 according to clinicopathological characteristics is shown in Table 1; in Figure 1 are presented representative pictures of prelamin A and RAD51 immunostaining in cancer specimens taken at the time of diagnosis. No association with age, grade of differentiation, FIGO stage, and tumor size was found for both biomarkers. Notably, the percentage of cases with a high prelamin A levels (score 3 and 4) was significantly greater in patients who did completely respond to treatment (pR0, 25 of 40, 63%) as compared to patients who did not respond (pR2, 6 of 26, 23%) (*p*=0.002) (Table 1). Moreover, cases with a high prelamin A were observed more frequently in patients showing absence of metastatic involvement of lymph nodes compared to patients with positive lymph node status after chemoradiation (56% *versus* 14%; *p*=0.0068). Accordingly, our results revealed an high RAD51 protein level (score 3 and 4) in non-responsive (pR2, 14 of 26, 53.8%) compared with responsive (pR0, 12 of 40, 30%) LACC patients, difference approaching statistical significance (*p*=0.072). On the other hand, RAD51 positivity was not associated to pathological lymph node status after chemoradiation.

**Table 1:** Prelamin A and RAD51 expression in the overall series

Characteristics	No. of patients	Prelamin A high N (%)	*p*^*a*^	RAD51 high N (%)	*p*^*a*^
**All cases**	66	31 (47.0)	-	26 (39.4)	-
**Age (years)**					
≤ 52	35	16 (45.7)	1	12 (34.3)	0.452
> 52	31	15 (48.4)		14 (45.2)	
**Grade**					
G1-G2	30	18 (60.0)	0.120	11 (36.7)	0.601
G3	30	11 (36.7)		14 (46.7)	
Not available	6	2		1	
**FIGO stage**					
IB2–II	56	27 (48.2)	0.739	22 (39.3)	1
III–IV	10	4 (40)		4 (40)	
**Tumor size (cm)**					
< 4	6	1 (16.7)	0.200	2 (33.3)	1
≥ 4	59	30 (50.8)		24 (40.7)	
Not available	1	-		-	
**Cervical residual tumor after CT/RT (mm)**					
pR0= 0	40	25 (62.5)	0.0024	12 (30.0)	0.072
pR2=5-80	26	6 (23.1)		14 (53.8)	
**Pathologic lymph node status after CT/RT**					
Negative	52	29 (55.8)	0.0068	20 (38.5)	0.768
Positive	14	2 (14.3)		6 (42.8)	

^*a*^ calculated by Fisher’s exact test. CT/RT = chemoradiation.

Protein expression levels were also measured by western blotting analysis in a limited case series including patients with complete response to neoadjuvant treatment (pR0) or macroscopic residual tumor (pR2) (n=10 for both groups, Figure 1B and 1F). Notably, results showed significantly higher prelamin A and lower RAD51 levels in pR0 compared to pR2 cervical tumors (*p*<0.05 for both, Figure 1C and 1G), and the Spearman’s Rank analysis showed a significant negative correlation between the level of the two biomarkers (r= -0.6, *p*<0.05). *LMNA* gene expression levels were also quantified by RT-qPCR and, as shown in Figure 1D, pR0 patients exhibited a tendency towards higher transcript levels compared to pR2, although difference did not achieve statistical significance (n=10 for both groups, *p*=0.4). Quantification of *RAD51* mRNA by RT-qPCR (Figure 1H) showed, in line with protein data, lower levels in patients exhibiting complete response to treatment (pR0) compared to patients with macroscopic residual tumor (pR2), difference approaching statistical significance (*p*=0.07).

### Predictors of residual disease after CT/RT

In the univariate analysis, low prelamin A and high RAD51 proved to be associated with poor chance of response to neoadjuvant treatment (complete response *versus* macroscopic response, Table 2). When logistic regression was applied, only low prelamin A retained an independent role in predicting a poor chance of response to treatment (*p*=0.003), while high RAD51 approached statistical significance (*p*=0.07) (Table 2).

**Table 2:** Univariate and multivariate analyses of clinicopathological parameters as predictors of pR2 after preoperative chemoradiation in LACC patients

Variables	Univariate		Multivariate	
	OR (95%CI)	*p*^*a*^	OR (95%CI)	*p*^*a*^
**Age (years)**				
≤ 52	0.95 (0.35-2.50)	1.00	-	-
> 52				
**Grade**				
G1-G2	2.3 (0.81-6.70)	0.19	-	-
G3				
**FIGO stage**				
IB2–II	2.7 (0.68-10.72)	0.17	-	-
III–IV				
**Tumor size (cm)**				
< 4	3.4 (0.38-31.24)	0.39	-	-
≥ 4				
**Prelamin A expression**				
Low	0.18 (0.06-0.55)	0.0024	0.18 (0.06-0.6)	0.003
High				
**RAD51 expression**				
Low	2.72 (0.98-7.6)	0.07	2.8 (0.9-8.6)	0.07
High				

^***a***^ only variables with *p* ≤ 0.1 at univariate analysis were included in the multivariate logistic regression model.

### Survival analysis

Follow-up data were available for all patients. As of January 2017, the median follow-up was 51 months (range, 5–134 months). During the follow-up period, recurrence and death were observed in 19 and 18 of 66 patients (28.8 % and 27.3%, respectively). The prognostic role of prelamin A and RAD51 expression for both progression free survival (PFS) and overall survival (OS) was tested in univariate and multivariate analyses, adjusted for clinicopathological parameters (Tables 3 and 4). No differences in terms of DFS and OS were documented according to the expression of prelamin A in LACC patients. On the other hand, univariate analysis demonstrated a significantly shorter PFS and OS in patients expressing high, compared to low, RAD51 protein levels (*p*=0.011 and *p*=0.05, respectively) (Tables 3 and 4, Figure 1I). As variables found to have prognostic influence in univariate analyses might covariate, all statistically significant variables from the univariate analysis were included in the multivariate regression analysis to identify independent prognostic factors. RAD51 expression proved to be an independent prognostic factor (*p*=0.038) for DFS, as did tumor stage and pathological response (*p*=0.04 and *p*=0.003, respectively) (Table 3). A decreased OS was also observed for patients with high RAD51 compared with low RAD51 expression, although difference only approached statistical significance (*p*=0.09). Other predictors of poor OS were advanced tumor stage and pathological response (*p*= 0.019 and *p*=0.011, respectively) (Table 4).

**Table 3:** Univariate and multivariate analysis of factors affecting DFS in LACC patients

Variables	Univariate		Multivariate	
	HR (95%CI)	p^*^	HR (95%CI)	p^*^
**Age (yrs)**				
≤ 52	1.22 (0.49-3.03)	0.663	-	-
> 52				
**Grade**				
G1-G2	2.30 (0.93-5.71)	0.071	1.71 (0.59-5.0)	0.326
G3				
**FIGO stage**				
IB–II	7.98 (1.9-33.61)	0.005	3.0 (1.03-8.88)	0.043
III–IV				
**Cervical residual tumor after CT/RT**				
pR0	9.52 (3.58-25.34)	<0.0001	7.88 (2.1-30.20)	0.003
pR2				
**Pathologic lymph nodal status after CT/RT**				
Negative	5.95 (1.79-19.80)	0.004	0.71 (0.23-2.17)	0.54
Positive				
**Prelamin A expression**				
Low	1.19 (0.48-2.96)	0.701	-	-
High				
**RAD51 expression**				
Low	3.43 (1.33-8.83)	0.011	2.9 (1.06-8.13)	0.038
High				

DFS = Disease-Free Survival. HR = Hazard ratio. CI = confidence interval. ^*^Ps were derived from the COX proportional hazards model. CT/RT = chemoradiation. Only variables with p-value ≤ 0.1 in the univariate analysis were included in multivariate model. χ^2^ of the model = 27.6; *p* value <0.0001.

**Table 4:** Univariate and multivariate analysis of factors affecting OS in LACC patients

Variables	Univariate		Multivariate	
	HR (95%CI)	p^*^	HR (95%CI)	p^*^
**Age (yrs)**				
≤ 52	1.52 (0.60-3.8)	0.379	-	-
> 52				
**Grade**				
G1-G2	1.41 (0.54-3.67)	0.481	-	-
G3				
**FIGO stage**				
IB–II	11.74 (2.57-53.71)	0.002	3.70 (1.24-11.04)	0.019
III–IV				
**Cervical residual tumor after CT/RT**				
pR0	10.66 (3.94-28.82)	<0.0001	6.17 (1.52-25.08)	0.011
pR2				
**Pathologic lymph nodal status after CT/RT**				
Negative	11.66 (3.34-40.72)	0.0001	1.92 (0.64-5.77)	0.24
Positive				
**Prelamin A expression**				
Low	1.08 (0.43-2.75)	0.868	-	-
High				
**RAD51 expression**				
Low	2.59 (1.00-6.70)	0.05	2.36 (0.86-6.49)	0.09
High				

OS = Overall Survival. HR = Hazard ratio. CI = confidence interval. ^*^Ps were derived from the COX proportional hazards model. CT/RT = chemoradiation. Only variables with p-value ≤ 0.1 in the univariate analysis were included in multivariate model. χ^2^ of the model = 28.7; *p* value <0.0001.

## DISCUSSION

To the best of our knowledge this is the first study focused on the concomitant evaluation of prelamin A and RAD51 as predictors of pathologic response in patients undergoing neoadjuvant chemoradiation therapy for locally advanced cervical cancer. Specifically, we found that the pathological tumor response to chemoradiation was significantly reduced in tumors with low- versus high- prelamin A level in pre-treatment biopsy tissues; likewise, high RAD51 was associated with a significantly lower rate of pathological complete response compared to tumors with low expression. Only prelamin A retained an independent predictive role after adjustment for relevant covariates, though RAD51 also approached statistical significance. If clinically validated in prospective studies, our data support a role for utilizing prelamin A and RAD51 as predictive biomarkers for the efficacy of neoadjuvant chemoradiation therapy in patients with cervical cancer.

Sustenance for our findings derives from evidences on the role of lamin A in cancer in general [22] and, more specifically, in cervical cancer. Indeed, besides studies linking lamin A/C downregulation to HPV infection [29], more recently, Capo-chichi and colleagues suggested that lamin A/C deficiency may serve as an independent risk factor for CIN development and as an indicator for preventive therapy in cervical cancer [30]. It is worthy to note, however, that previous studies investigating the role of lamin A/C in cervical cancer actually used antibodies that did not distinguish between prelamin A and mature lamin A [29, 30]; conversely, we selected an anti-prelamin A polyclonal antibody that detects full-length prelamin A which has not yet undergone to proteolytic steps (both in its non-farnesylated and in its farnesylated form), while not cross-reacting with mature lamin A. In this respect, our results may provide important insights into the mechanisms underlying disease development. Several putative molecular mechanisms may be involved in A-type lamins alterations including mutations in the *LMNA* gene [20], epigenetic *LMNA* promoter modifications [31], degradation induced by viral infection (either HPV or HIV) [29], or impaired activity of a protease (ZMPSTE24) involved in the maturation of prelamin A into functional lamin A [32, 33]. Although deeper and more comprehensive validation analyses with functional assays are needed before any definite conclusion can be drawn, overall our findings suggest that in LACC patients with no responsiveness to CT/RT, prelamin A–processing might be more efficient and lamin A possibly more actively produced.

It is well known that several layers of regulation in the DNA damage response intersect with lamin: loss of lamin A function reduces the transcription of RAD51 and BRCA1 and also results in the downregulation and mislocalization of the tumor suppressors RB105 and ING1, as well as the DNA repair factor p53-binding protein 1 (53BP1) [22]. These types of target alterations can ultimately promote sensitivity to chemotherapeutic strategies that capitalize on an impaired ability for DNA repair, including cisplatin and radiation. RAD51 represents a central protein of homologous recombination (HR) pathway in response to DSBs induced by radiation and/or chemotherapy treatment such as cisplatin, with several studies showing that overexpression of RAD51 is associated with treatment resistance and outcome in a variety of tumors [34]. Here we show indeed that RAD51 overexpression is associated with reduced response to therapy and an unfavorable outcome, emerging as an independent prognostic factor for disease free survival, and approaching statistical significance for overall survival. Notably, this trend was confirmed by WB analysis in a subset of patients, and, more importantly, we found, in the same patients, a upregulation in *RAD51* mRNA levels, as expected on the basis of previous literature data. In line with our results, previous studies demonstrated increased RAD51 expression in pre-treatment tumor biopsies in non-responsive compared with responsive advanced squamous cervical cancer patients [35]. Besides, a protective effect on clinical outcome of cervical cancer patients of the *RAD51 172TT* genotype, has been also reported as a possible consequence of a lower cellular capacity for DSB repair and, in turn, a greater sensitivity to chemotherapeutic agents [36]. It is worthy to note that inhibiting RAD51 has been explored as a way to sensitize cancer cells to chemotherapy and radiotherapy and, more recently, the benefits of targeting RAD51 in combination with conventional cancer therapies and newer PARPi [poly (ADP-ribose) polymerase inhibitors] treatments have been described [34].

The lack of any prognostic role for prelamin A expression remains to be clarified: indeed, besides the relatively small sample size of our series, also the complexity of this biologic pathway may result in heterogeneity of clinical data. In addition, it has to be acknowledged that the association between biological markers and response to local treatments, such as pelvic CT/RT, does not imply *tout court* the association with survival outcome, as well as pattern of relapse (e.g. local, loco-regional, distant).

In conclusion, our findings, that should be validated in a larger prospective trial, provide a framework for sustaining molecular-based patient-tailored treatments, employing RAD51 inhibitors as single agents or in combination with other therapies for cervical cancer.

## MATERIALS AND METHODS

### Patients

This retrospective study included 66 cervical cancer patients admitted to the Gynecologic Oncology Unit, Catholic University of the Sacred Heart, Rome between April 2001 and April 2013. Staging was performed according to FIGO classification. Patients with a diagnosis of stage IB2-IVA LACC disease were evaluated in the study. The trial was approved by the local Ethics Committee and Institutional Review Board (Protocol P/966/CE/2012) and all patients signed a written informed consent agreeing to submit to all the procedures described and for their data to be collected. Pre-treatment tumour tissue biopsies were obtained during staging procedures, the joint assessment by surgeon and pathologist allowing an unequivocal identification of tumor area to be sampled. Tissue specimens were formalin-fixed paraffin-embedded for diagnostic histopathology and immunohistochemical analyses. For a subset of patients, frozen tissue samples were also available for analysis (n=20). Patients received preoperative CT/RT; RT was administered to the pelvic region (39.6-50.3 Gy) according to specific protocols, and concomitant chemotherapy included cisplatin and 5-fluorouracil or capecitabine [9, 10]. Seven or 8 weeks after the end of concomitant CT/RT, all cases were submitted to radical hysterectomy and pelvic ± aortic lymphadenectomy. After surgery, patients were triaged to routine follow-up procedure according to the previously reported schedule [9, 10]. Pathologic complete response was defined as the absence of any residual tumor after treatment at any site level (residual tumor=None, pR0), microscopic response included cases with persistence of only microscopic tumor foci at any site level (≤ 3 mm maximum dimension, pR1), while macroscopic response included cases with persistence of residual tumor > 3 mm (maximum dimension, pR2) [37]. In order to maximize the identification of potential differences in the biomarker profile associated with CT/RT responsiveness, we decided to focus our analysis on patients with complete response *versus* macroscopic residual tumor. The clinicopathological characteristics of the study population have been summarized in Table 5. Histopathologically normal cervical samples (n=5), as well as low-grade (low-SIL, n=5), and high-grade squamous intraepithelial lesion (high-SIL, n=5) were also included in the study.

**Table 5:** Clinicopathological features of the overall series

Characteristics	No. of patients (%)
**All cases**	66
**Age (years)**	
≤ 52	35 (53.0)
> 52	31 (47.0)
**Histotype**	
Squamous	64 (97)
Other	2 (3)
**Grade**	
1-2	30 (45.5)
3	30 (45.5)
Not available	6 (9.0)
**FIGO stage**	
IB2–II	56 (84.8)
III–IV	10 (15.2)
**Tumor size (cm)**	
< 4	6 (9.1)
≥ 4	59 (89.4)
Not available	1 (1.5)
**Cervical residual tumor after CT/RT (mm)**	
pR0 = 0	40 (60.6)
pR2 = 5-80 (median value 17)	26 (39.4)
**Pathologic lymph node status after CT/RT**	
Negative	52 (78.8)
Positive	14 (21.2)

CT/RT = chemoradiation.

### Immunohistochemical analyses

Immunohistochemical analysis was carried out on three-micrometer-thick paraffin sections as previously described [38]. Briefly, 3-μm thick tumor sections were de-waxed and rehydrated; heat-induced antigen retrieval was carried out using EDTA buffer (pH 8.0) for 30 minutes, then to inhibit endogenous peroxidase the sections were incubated in 3% H_2_O_2_ in distillate water for 10 minutes. Sections were incubated with primary antibody at dilution 1:100, overnight at 4°C for rabbit anti-human Prelamin A (≠0045, Diatheva Cartoceto PU, Italy), and at 1h at 37°C for mouse anti-human RAD51 [≠MA5-14419, clone 51RAD01(3C10), Invitrogen Carlsbad, California, USA]. Sections were incubated with the secondary anti-rabbit EnVision System-HRP (DakoCytomation Carpinteria, CA, USA) against Prelamin A and anti-mouse EnVision System-HRP (DakoCytomation) against RAD51. The analysis of all tissue sections was done without any prior knowledge of clinical parameters by 3 authors (SL, MM, IDS) by means of light microscopy. For both proteins, staining intensity was estimated by: *i*) a four-step scale based on visual examination; *ii*) the percentage of positive cells from 0 to 100%. Using these two parameters (intensity and % positive tumor cells) a final IHC score was composed according to the following criteria: negative= tumors without any detectable staining; score 1= tumors with very low staining intensity in < 80% tumor cells; score 2= tumors with low staining intensity in <50% tumor cells; score 3= tumors with high staining intensity in >50% tumor cells; score 4= tumors with very high staining intensity in >80% tumor cells. The proportion of immunostained tumor cells was assessed by evaluating the entire tumor area. Tumors with score 1 and 2 were rated as “low” whereas those with score 3 and 4 were rated as “high”. Cut-off values were defined according to data distribution, showing that in the entire cancer series prelamin A and RAD51 levels were 2.2 ± 0.14 and 2.4 ± 0.12, respectively.

### Western blot analysis

Protein were isolated from frozen tissue biopsies using the All Prep^®^ DNA/RNA/Protein Mini Kit (Qiagen, Germany) following the manufacturer’s protocol. After extraction, equal amounts of protein (30 μg/sample) were separated using a 10% precast gel (Mini-PROTEAN® TGX™ Precast Protein Gel Bio-rad Laboratories Hercules, California), subsequently blotted to PVDF mini-membrane (Trans-Blot® Turbo™ Mini PVDF Transferand) and transferred using the Trans-Blot® Turbo™ Transfer System (Bio-Rad) with 25V, 1.0 A, for 30 minutes. Sequentially the membrane was probed with the following primary antibodies: anti-prelamin A dilution 1:1000 (Diatheva); anti-RAD51 dilution 1:500 (Invitrogen); anti-HSP70 (cod H5147, Sigma-Aldrich Saint Louis USA) at 4°C overnight. Specific proteins were visualized with Image- Quant LAS 500 system (GE Healthcare Europe GmbH, Milan, Italy).

### Real time PCR

Total RNA was extracted from frozen tissue biopsies using AllPrep DNA/RNA/Protein Mini Kit (Qiagen). RNA concentration was measured using a NanoDrop 2000 spectrophotometer (Thermo Scientific, Waltham, MA) and RNA integrity was confirmed by Bioanalyzer (Agilent Technologies, Palo Alto, CA). All samples had an RNA integrity number (RIN) in the range 4.0–10.0. RNA was reverse-transcribed to cDNA using iScript™ cDNA Synthesis Kit (Bio-Rad), according to the manufacturer’s protocol. To evaluate *RAD51* and *LMNA* mRNA levels, each cDNA was subjected to real time PCR (qPCR). Amplifications were carried out using specific primers and the iQ SYBR Green Supermix (Bio-Rad) in a final volume of 20 μl, starting with a 3-min template denaturation step at 95°C followed by 40 cycles of 15 s at 95°C and 1 min at 60°C. RT-qPCR was carried out in CFX Connect Real Time PCR Detection System (Bio-Rad), according to manufacturer’s instructions. In each assay, standard curves were generated using a serial dilution of the initial amount of control cDNA to determine the range of template concentrations, which showed a good linearity and efficiency for the different reactions; efficiency values between 80 and 100% were found for each primer set and taken into account for the comparative quantitation analysis. Melting curves of the reaction products were also generated to assess the specificity of the measured fluorescence. The PCR primers for detecting specific transcripts were the following: *LMNA* forward CGGATGCGCTGCAGGAA; reverse CCAGGTTGCTGTTCCTCTCA; *RAD51* forward 5′-AGCTGGGAACTGCAACTCAT-3′; reverse 5′-CTGCATTGCCATTAGCTCCAC-3′; *ACTB* forward CCAACCGCGAGAAGATGAC; reverse TAGCACAGCCTGGATAGCAA; *B2M* forward 5′- TTAGCTGTGCTCGCGCTAC-3′; reverse 5′-CTCTGCTGGATGACGTGAGTAA-3′; *GAPDH* forward GAACGGGAAGCTTGTCATCAA; reverse ATCGCCCCACTTGATTTTGG. Among the endogenous reference genes included on the array (*GAPDH*, *ACTB*, *B2M*) *B2M* was chosen for normalizing the data following visualization of the global Ct value distribution. All samples were amplified in triplicate and normalized to the housekeeping gene, *B2M*. The mean of threshold cycles (Ct, take-off point of reactions) for each specimen was used to obtain the fold change of gene expression level according to the -ΔΔCt method [39] and relative to the mean ΔCt of the whole population of samples.

### Statistical analysis

The expression of molecular biomarkers and their association according to clinicopathological parameters were evaluated using the Fisher’s exact test. A logistic regression model was used to investigate the role of clinicopathological variables as predictors of pathological response after CT/RT. The prognostic effect of the various parameters on clinical outcome (i.e. recurrence or death of disease) was tested by plotting survival curves according to Kaplan–Meier method, and comparing groups using the log rank test, as well as by multivariate analysis using the Cox model. Kaplan–Meier survival estimates were generated from date of histological diagnosis to time of the last follow up or death. In univariate analysis, each parameter was categorized for subsequent statistical analysis. Only variables with p-value ≤0.1 in the univariate analysis were included in multivariate model. P values were two-sided, with *p*≤0.05 considered as significant. The remaining data were analyzed using the Mann–Whitney U test. Correlations between variables were identified employing the Spearman’s rank correlation. All statistical analyses were performed using the GraphPad Prism5 Software (San Diego, CA, USA). Cox analysis was performed using the StatPlus 2009 (AnalystSoft, Vancouver, Canada).

## SUPPLEMENTARY MATERIALS FIGURE



## References

[1] (January 23, 2017). GLOBOCAN 2012 (IARC). Section of Cancer Information.

[2] Burd EM (2003). Human Papillomavirus and Cervical Cancer. Clin Microbiol Rev.

[3] Monk BJ, Tewari KS, Koh WJ (2007). Multimodality therapy for locally advanced cervical carcinoma: state of the art and future directions. J Clin Oncol.

[4] Gaffney DK, Soisson AP (2010). Simple or complex: Optimal therapy for cancer of the cervix. Gynecol Oncol.

[5] Eifel PJ, Winter K, Morris M, Levenback C, Grigsby PW, Cooper J, Rotman M, Gershenson D, Mutch DG (2004). Pelvic irradiation with concurrent chemotherapy versus pelvic and para-aortic irradiation for high-risk cervical cancer: an update of radiation therapy oncology group trial (RTOG) 90-01. J Clin Oncol.

[6] Chemoradiotherapy for Cervical Cancer Meta-analysis Collaboration (CCCMAC) (2010). Reducing uncertainties about the effects of chemoradiotherapy for cervical cancer: individual patient data meta-analysis. Cochrane Database Syst Rev.

[7] Noordhuis MG, Eijsink JJ, Roossink F, de Graeff P, Pras E, Schuuring E, Wisman GB, de Bock GH, van der Zee AG (2011). Prognostic cell biological markers in cervical cancer patients primarily treated with (chemo)radiation: a systematic review. Int J Radiat Oncol Biol Phys.

[8] Classe JM, Rauch P, Rodier JF, Morice P, Stoeckle E, Lasry S, Houvenaeghel G, Groupe des Chirurgiens de Centre de Lutte Contre le Cancer (2006). Surgery after concurrent chemoradiotherapy and brachytherapy for the treatment of advanced cervical cancer: morbidity and outcome: results of a multicenter study of the GCCLCC (Groupe des Chirurgiens de Centre de Lutte Contre le Cancer). Gynecol Oncol.

[9] Ferrandina G, Legge F, Fagotti A, Fanfani F, Distefano M, Morganti A, Cellini N, Scambia G (2007). Preoperative concomitant chemoradiotherapy in locally advanced cervical cancer: safety, outcome and prognostic measures. Gynecol Oncol.

[10] Ferrandina G, Margariti PA, Smaniotto D, Petrillo M, Salerno MG, Fagotti A, Macchia G, Morganti AG, Cellini N, Scambia G (2010). Long-term analysis of clinical outcome and complications in locally advanced cervical cancer patients administered concomitant chemoradiation followed by radical surgery. Gynecol Oncol.

[11] Motton S, Houvenaeghel G, Delannes M, Querleu D, Soulé-Tholy M, Hoff J, Lèguevaque P (2010). Results of surgery after concurrent chemoradiotherapy in advanced cervical cancer: comparison of extended hysterectomy and extrafascial hysterectomy. Int J Gynecol Cancer.

[12] Ferrandina G, Ercoli A, Fagotti A, Fanfani F, Gallotta V, Margariti AP, Salerno MG, Chiantera V, Legge F, Macchia G, Morganti AG, Valentini V, Scambia G (2014). Completion surgery after concomitant chemoradiation in locally advanced cervical cancer: a comprehensive analysis of pattern of postoperative complications. Ann Surg Oncol.

[13] Ferrandina G, Gambacorta A, Gallotta V, Smaniotto D, Fagotti A, Tagliaferri L, Foti E, Fanfani F, Autorino R, Scambia G, Valentini V (2014). Chemoradiation with concomitant boosts followed by radical surgery in locally advanced cervical cancer: long-term results of the ROMA-2 prospective phase 2 study. Int J Radiat Oncol Biol Phys.

[14] Morice P, Rouanet P, Rey A, Romestaing P, Houvenaeghel G, Boulanger JC, Leveque J, Cowen D, Mathevet P, Malhaire JP, Magnin G, Fondrinier E, Berille J, Haie-Meder C (2012). Results of the GYNECO 02 study, an FNCLCC Phase III trial comparing hysterectomy with no hysterectomy in patients with a (clinical and radiological) complete respose after chemoradiation therapy for stage IB” or II cervical cancer. Oncologist.

[15] Cetina L, González-Enciso A, Cantú D, Coronel J, Pérez-Montiel D, Hinojosa J, Serrano A, Rivera L, Poitevin A, Mota A, Trejo E, Montalvo G, Muñoz D (2013). Brachytherapy versus radical hysterectomy after external beam chemoradiation with gemcitabine plus cisplatin: a randomized, phase III study in IB2-IIB cervical cancer patients. Ann Oncol.

[16] Ferrandina G, Petrillo M, Restaino G, Rufini V, Macchia G, Carbone A, Zannoni GF, Lucidi A, D’Angelo G, Scambia G (2012). Can radicality of surgery be safely modulated on the basis of MRI and PET/CT imaging in locally advanced cervical cancer patients administered preoperative treatment?. Cancer.

[17] Kitahara O, Katagiri T, Tsunoda T, Harima Y, Nakamura Y (2002). Classification of sensitivity or resistance of cervical cancers to ionizing radiation according to expression profiles of 62 genes selected by cDNA microarray analysis. Neoplasia.

[18] Harima Y, Ikeda K, Utsunomiya K, Shiga T, Komemushi A, Kojima H, Nomura M, Kamata M, Sawada S (2009). Identification of genes associated with progression and metastasis of advanced cervical cancers after radiotherapy by cDNA microarray analysis. Int J Radiat Oncol Biol Phys.

[19] Schreiber KH, Kennedy BK (2013). When lamins go bad: nuclear structure and disease. Cell.

[20] Musich PR, Zou Y (2011). DNA-damage accumulation and replicative arrest in Hutchinson-Gilford progeria syndrome. Biochem Soc Trans.

[21] di Masi A, D’Apice MR, Ricordy R, Tanzarella C, Novelli G (2008). The R527H mutation in LMNA gene causes an increased sensitivity to ionizing radiation. Cell Cycle.

[22] Chow KH, Factor RE, Ullman KS (2012). The nuclear envelope environment and its cancer connections. Nat Rev Cancer.

[23] Broers JL, Machiels BM, Kuijpers HJ, Smedts F, van den Kieboom R, Raymond Y, Ramaekers FC (1997). A- and B-type lamins are differentially expressed in normal human tissues. Histochem Cell Biol.

[24] Higareda-Almaraz JC, Enríquez-Gasca Mdel R, Hernández-Ortiz M, Resendis-Antonio O, Encarnación-Guevara S (2011). Proteomic patterns of cervical cancer cell lines, a network perspective. BMC Syst Biol.

[25] Redwood AB, Gonzalez-Suarez I, Gonzalo S (2011). Regulating the levels of key factors in cell cycle and DNA repair: new pathways revealed by lamins. Cell Cycle.

[26] Chappell WH, Gautam D, Ok ST, Johnson BA, Anacker DC, Moody CA (2016). Homologous recombination repair factors Rad51 and BRCA1 are necessary for productive replication of human papillomavirus 31. J Virol.

[27] Zempolich K, Fuhrman C, Milash B, Flinner R, Greven K, Ryu J, Forbes A, Kerlin K, Nichols RC, Gaffney DK (2008). Changes in gene expression induced by chemoradiation in advanced cervical carcinoma: a microarray study of RTOG C-0128. Gynecol Oncol.

[28] Raderschall E, Bazarov A, Cao J, Lurz R, Smith A, Mann W, Ropers HH, Sedivy JM, Golub EI, Fritz E, Haaf T (2002). Formation of higher-order nuclear Rad51 structures is functionally linked to p21 expression and protection from DNA damage-induced apoptosis. J Cell Sci.

[29] Kivi N, Greco D, Auvinen P, Auvinen E (2008). Genes involved in cell adhesion, cell motility and mitogenic signaling are altered due to HPV 16 E5 protein expression. Oncogene.

[30] Capo-chichi CD, Aguida B, Chabi NW, Cai QK, Offrin G, Agossou VK, Sanni A, Xu XX (2016). Lamin A/C deficiency is an independent risk factor for cervical cancer. Cell Oncol (Dordr).

[31] Agrelo R, Setien F, Espada J, Artiga MJ, Rodriguez M, Pérez-Rosado A, Sanchez-Aguilera A, Fraga MF, Piris MA, Esteller M (2005). Inactivation of the lamin A/C gene by CpG island promoter hypermethylation in hematologic malignancies, and its association with poor survival in nodal diffuse large B-cell lymphoma. J Clin Oncol.

[32] Coffinier C, Hudon SE, Farber EA, Chang SY, Hrycyna CA, Young SG, Fong LG (2007). HIV protease inhibitors block the zinc metalloproteinase ZMPSTE24 and lead to an accumulation of prelamin A in cells. Proc Natl Acad Sci U S A.

[33] Liu Q, Kim DI, Syme J, LuValle P, Burke B, Roux KJ (2010). Dynamics of lamin-A processing following precursor accumulation. PLoS One.

[34] Ward A, Khanna KK, Wiegmans AP (2015). Targeting homologous recombination, new pre-clinical and clinical therapeutic combinations inhibiting RAD51. Cancer Treat Rev.

[35] Balacescu O, Balacescu L, Tudoran O, Todor N, Rus M, Buiga R, Susman S, Fetica B, Pop L, Maja L, Visan S, Ordeanu C, Berindan-Neagoe I, Nagy V (2014). Gene expression profiling reveals activation of the FA/BRCA pathway in advanced squamous cervical cancer with intrinsic resistance and therapy failure. BMC Cancer.

[36] Nogueira A, Catarino R, Faustino I, Nogueira-Silva C, Figueiredo T, Lombo L, Hilário-Silva I, Pereira D, Medeiros R (2012). Role of the RAD51 G172T polymorphism in the clinical outcome of cervical cancer patients under concomitant chemoradiotherapy. Gene.

[37] Zannoni GF, Vellone VG, Carbone A (2008). Morphological effects of radiochemotherapy on cervical carcinoma: a morphological study of 50 cases of hysterectomy specimens after neoadjuvant treatment. Int J Gynecol Pathol.

[38] Zannoni GF, Prisco MG, Vellone VG, De Stefano I, Scambia G, Gallo D (2011). Changes in the expression of oestrogen receptors and E-cadherin as molecular markers of progression from normal epithelium to invasive cancer in elderly patients with vulvar squamous cell carcinoma. Histopathology.

[39] Livak KJ, Schmittgen TD (2001). Analysis of relative gene expression data using real-time quantitative PCR and the 2(-Delta Delta C(T)) method. Methods.

