# Supplementary material for: The relevance of prelamin A and RAD51 as molecular biomarkers in cervical cancer
Source: Oncotarget. 2017 Oct 9;8(55):94247–58. doi: 10.18632/oncotarget.21686 (PMC5706871; doi:10.18632/oncotarget.21686)
Supplement: Supplementary file 1 [file oncotarget-08-94247-s001.pdf]

The relevance of prelamin A and RAD51 as molecular biomarkers in cervical cancer

SUPPLEMENTARY MATERIALS

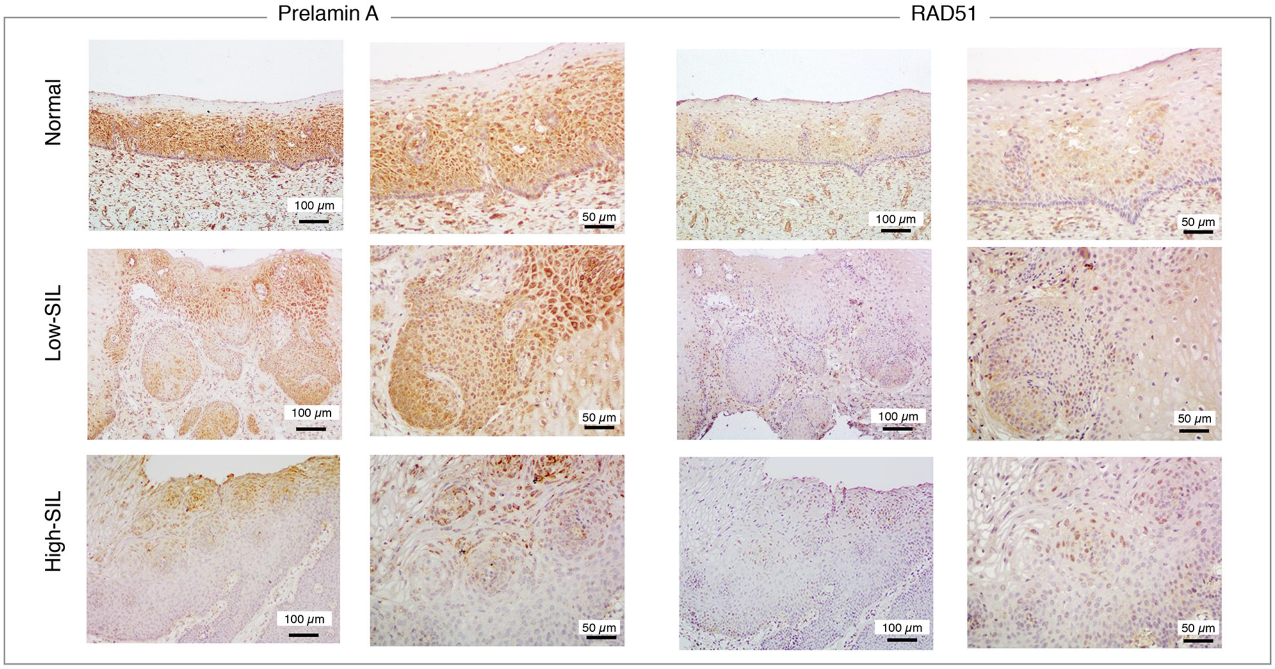

Supplementary Figure 1: Representative pictures for prelamin A and RAD51 immunostaining in normal cervical epithelium, low-SIL and high-SIL (magnification 10x and 20x).
